# Improving Precision Force Control With Low-Frequency Error Amplification Feedback: Behavioral and Neurophysiological Mechanisms

**DOI:** 10.3389/fphys.2019.00131

**Published:** 2019-02-20

**Authors:** Ing-Shiou Hwang, Chia-Ling Hu, Zong-Ru Yang, Yen-Ting Lin, Yi-Ching Chen

**Affiliations:** ^1^Institute of Allied Health Sciences, College of Medicine, National Cheng Kung University, Tainan, Taiwan; ^2^Department of Physical Therapy, College of Medicine, National Cheng Kung University, Tainan, Taiwan; ^3^Physical Education Office, Asian University, Taichung, Taiwan; ^4^Department of Physical Therapy, College of Medical Science and Technology, Chung Shan Medical University, Taichung, Taiwan; ^5^Physical Therapy Room, Chung Shan Medical University Hospital, Taichung, Taiwan

**Keywords:** force fluctuations, stochastic processes, visuomotor, EMG, EEG

## Abstract

Although error amplification (EA) feedback has been shown to improve performance on visuomotor tasks, the challenge of EA is that it concurrently magnifies task-irrelevant information that may impair visuomotor control. The purpose of this study was to improve the force control in a static task by preclusion of high-oscillatory components in EA feedback that cannot be timely used for error correction by the visuomotor system. Along with motor unit behaviors and corticomuscular coherence, force fluctuations (Fc) were modeled with non-linear SDA to contrast the reliance of the feedback process and underlying neurophysiological mechanisms by using real feedback, EA, and low-frequency error amplification (LF-EA). During the static force task in the experiment, the EA feedback virtually potentiated the size of visual error, whereas the LF-EA did not channel high-frequency errors above 0.8 Hz into the amplification process. The results showed that task accuracy was greater with the LF-EA than with the real and EA feedback modes, and that LF-EA led to smaller and more complex Fc. LF-EA generally led to smaller SDA variables of Fc (critical time points, critical point of Fc, the short-term effective diffusion coefficient, and short-term exponent scaling) than did real feedback and EA. The use of LF-EA feedback increased the irregularity of the ISIs of MUs but decreased the RMS of the mean discharge rate, estimated with pooled MU spike trains. Beta-range EEG–EMG coherence spectra (13–35 Hz) in the LF-EA condition were the greatest among the three feedback conditions. In summary, amplification of low-frequency errors improves force control by shifting the relative significances of the feedforward and feedback processes. The functional benefit arises from the increase in the common descending drive to promote a stable state of MU discharges.

## Introduction

A general outcome of motor control is variability (Frank et al., 2006; Bays and Wolpert, 2007). The structures of movement variability [such as force fluctuations (Fc)] are not necessarily a direct consequence of neural noises. Contrary to the whiteness assumption, Fc are colored time series contingent upon environmental contexts and task demands (Miall et al., 1986, 1993). Fc are composed of numerous centrally scaled pulse-like elements that remedy tracking deviations during a visuomotor task (Navas and Stark, 1968; Miall et al., 1986; Slifkin et al., 2000; King and Newell, 2015). The spatial and temporal information in visual feedback determines the transitions of the motor state with respect to target constraints (Hwang et al., 2013; Chen et al., 2017b). Hence, Fc with visual feedback are smaller and have greater complexity as compared to those in a no-vision condition (Baweja et al., 2009). Force tracking results in higher complexity of Fc when the visual display has high spatial resolution than when it has low spatial resolution (Sosnoff et al., 2006). The reason is that high-sensitivity feedback with precise visual information can facilitate richer error correction strategies. A major determinant of Fc is variations in the discharge properties of MUs. In addition, corticomuscular coherence (EEG–EMG coherence) in the beta range (13–35 Hz) plays a critical role in stabilizing corticospinal communication during static contraction (Kristeva et al., 2007; Omlor et al., 2011). Greater beta EEG–EMG coherence represents more effective sensorimotor integration and greater attentional focus being directed toward stabilizing the force output (Witte et al., 2007).

Accurate visual feedback is important to develop a reliable perception–action link. Interestingly, visual display of performance outcomes that are worse than the actual performance can better expedite motor adaptations to novel task constraints than can accurate visual feedback (Patton et al., 2006; Domingo and Ferris, 2010; Reisman et al., 2013). The virtual amplification of task errors, or EA, is frequently used in combination with robotic technology to facilitate motor recovery in patients with neurological disorders (Abdollahi et al., 2014; Kao et al., 2015; Israely and Carmeli, 2016; Bouchard et al., 2017). EA is thought to inflate response conflicts in the error-monitoring network such that participants are more attentive to execution of the motor task (Boussaoud and Kermadi, 1997; Jueptner and Weiller, 1998; Shirzad and Van der Loos, 2012). Alternatively, a model-based study predicted that EA could minimize the effect of overt task fluctuations by reducing the neuromotor noise variance (Hasson et al., 2016). In addition to task improvement, a force-tracking task with EA leads to smaller Fc with higher spectral components and complexity (Williams et al., 2016; Chen et al., 2017b; Hwang et al., 2017). These scenarios support the potential functional benefits of visual EA, including deliberate and richer tuning behaviors with more frequent corrective attempts than with real visual feedback. Physiologically, visually exaggerated mismatches with visual EA favors the use of a feedback process to regulate the MU discharge and the variability of the ISI among those MUs (Chen et al., 2017b). However, the use of visual EA does not always result in behavior success (Wei et al., 2005; Sung and O’Malley, 2011; Bouchard et al., 2015). For instance, EA may add to perceptual conflicts among the visual, proprioceptive, and haptic inputs due to the distortion of real visual consequences (Ogawa and Imamizu, 2013). Moreover, EA may augment the visual information load by proportionately amplifying the full spectrum of execution errors, including functionally irrelevant visual stimuli that could impair the efficacy of corrective behaviors (Lipowski, 1975; Chen et al., 2017a). Hence, to optimize visual EA, it is necessary to focus on the usability of task-related information.

Given the potential positive effects with EA, this study aimed to contrast EA with and without high-frequency error components during low-level static contraction. We argue that not all of the error information, especially the fast-oscillatory components, is helpful to improve visuomotor performance. As a visuomotor task with EA favors the use of a feedback mechanism (Chen et al., 2017b), the amplified fast-oscillatory error components (>0.8 Hz) within visual feedback cannot be effectively used due to a significant delay of 150 ms in the visuomotor loop (Miall et al., 1985, 1986, 1993). The amplification of these high-frequency error components could offset the positive effect of EA on a visuomotor task. Only LF-EA, wherein the error information of rapid fluctuations is excluded, could increase the effectiveness of visual feedback (or a feedback-prone process) for corrections of force-tracking deviations. Employing non-linear Fc dynamics and mathematical decomposition of surface electromyography, this study contrasted the behavior and neural mechanisms of static force-tracking in the real, EA, and LF-EA feedback conditions. Due to potential changes in force gradation strategies, it was hypothesized that (1) the size, complexity, and SDA variables of Fc would be different in the three visual feedback modes (traditional visual feedback, EA, and LF-EA), and (2) the variations in MU discharge and central drive to stabilize corticomuscular communication would vary among the visual feedback modes.

## Materials and Methods

### Subjects

The participants were 15 healthy adults (8 males and 7 females; mean age: 24.8 ± 0.9 years, range: 21–31 years old) from a university campus or the local community. All were self-reported as being right-handed, and none had symptoms or signs of neuromuscular diseases. The experiments were conducted in accordance with the Declaration of Helsinki and approved by an authorized institutional human research review board (IRB) at the University Hospital of the Chung Shan Medical University, Taiwan. All participants signed a written informed consent form prior to inclusion.

### Experimental Procedures

**FIGURE 1:**
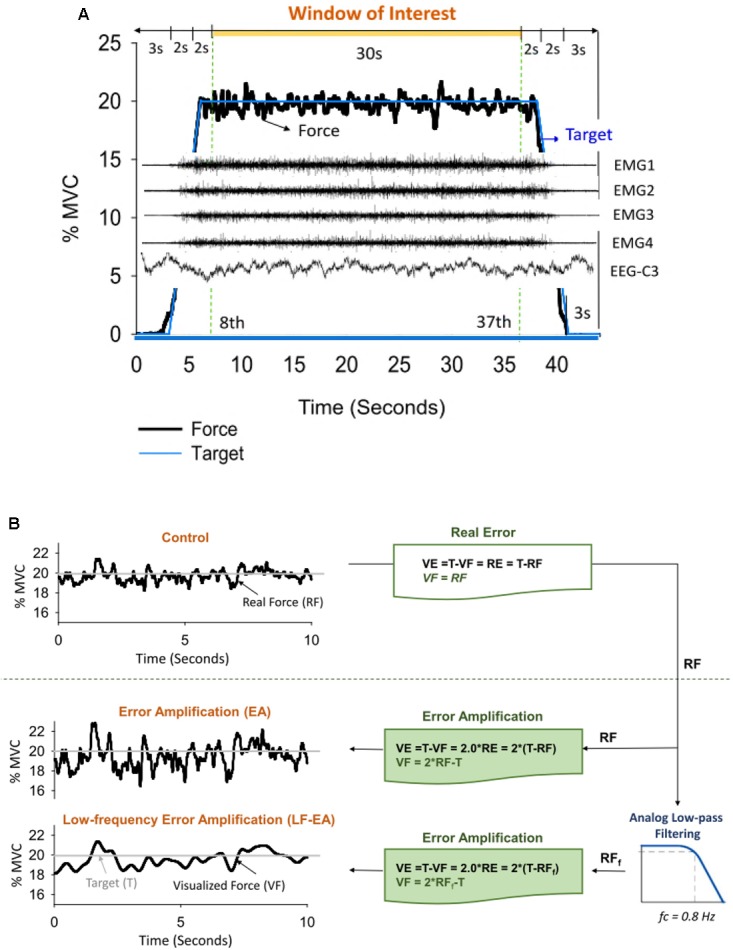
**(A)** Target signal and representative data. Only force data, EMG, and EEG in the time window of interest were presumably stable for subsequent feature extraction. **(B)** Illustration of manipulation of error augmentation for visual feedback. In the control condition, real force (RF) was shown on the monitor to guide the force task, so that real error (RE) is equivalent to visualized error (VE) during tracking. In the error amplification (EA) and low-frequency error amplification (LF-EA) conditions, the VE of the tracking task were magnified with mathematical transformation. The visualized force outputs (VF) in the EA condition represent on-line force feedback information that doubles the size of the execution error during force-tracking. The LF-EA feedback consists of a low-pass filtering process and an error amplification process during force-tracking. The low-pass filtering process suppresses high-frequency components (>0.8 Hz) of real force. Error amplification process based on the filtered real force (RF_f_) magnifies VE that contains only low-frequency components (<0.8 Hz).

The participants completed a unilateral static force task of isometric index abduction at a low force level (20% MVC) under three error feedback conditions: control, error-amplification (EA), and low-frequency error-amplification (LF-EA). The participants were seated with the palm and forearm of the right hand firmly fixed within a thermoplastic splint on the table. The index finger was held slightly abducted (5 degrees of abduction), and its abduction force was measured using a force transducer (Model: MB-100, Interface Inc., United States) followed by an analog amplifier (gain = 10). The cut-off frequency of the amplifier was 20 Hz so that fast-oscillatory force components such as 8–12 Hz physiological tremor would not be attenuated by the experiment setting. For each individual, the MVC of the FDI was pre-determined from three maximal contraction trials of 3 s separated by 3 min pauses, by averaging the largest force produced in each trial. Interleaved with 3-min pauses, separate experimental trials in the control, EA, and LF-EA conditions commenced in a randomized order, after three practice trials in all conditions. There were four experimental trials for the control, EA, or LF-EA conditions. During the force-tracking in the control condition, the participants were given 2 s to reach the target force (slope: 10% MVC/second) after a latent period of 3 s (Figure 1A). Then they coupled isometric force to the target signal (20% MVC) as precisely as possible by pushing their index finger against the force transducer for another 34 s under visual guidance. The force output returned to the resting level in 2 s, followed by a 3-s latency period. The time window of interest was denoted as the 8th to 37th seconds in a total of 44 s for an experimental trial. The resolution of the display of visual feedback on the monitor was 1,920 pixels × 1,080 pixels.

In the EA condition, the VF displayed on the monitor was mathematically transformed to potentiate execution error [mismatches between the real force output (RF) and the target signal (T)] (Figure 1B). The VF was equal to the sum of twice the RF minus the target signal (T) (VF = 2RF-T), so the participant would perceive twice the amount of the RE of the static force-tracking task (VE = 2RE). RF in the EA condition was low-pass filtered at 20 Hz, and the VF was relatively noisy, containing enhanced fast-oscillatory force components and tremulous movements. In the LF-EA condition, the RF came from a parallel force channel that pre-conditioned the force output with an analog low-pass filter (cut-off frequency: 0.8 Hz) prior to amplification (Figure 1B). The VF was much smoother in the LF-EA condition than in the EA condition. The participants could hardly correct high-frequency errors above 0.8 Hz via visual feedback (Pew, 1974; Miall et al., 1985), because the time period between the pick-up of visual information and its use in producing a required adjustment was at least 150 ms (Miall et al., 1986). For all the feedback conditions, the spatial gain to display the target signal and the force output was roughly 25 pixels per 1% MVC. The inter-trial interval of rest was 2 min. In the LabVIEW platform (LabVIEW v.8.5, National Instruments Inc., United States), the RF conditioned with a low-pass filter at 20 Hz and the target signal were digitalized at 1 kHz by a 16-bit analog-to-digital converter (DAQCard-6024E; National Instruments Inc., United States) in the EA and LF-EA conditions. For the LF-EA condition, the smoother force channel, conditioned with an analog low-pass filter (cut-off frequency: 0.8 Hz), was also recorded.

### Electromyographic and Electroencephalographic Recordings

In addition to the force signal, we synchronized multi-electrode surface EMG with 5 surface pin-sensors (0.5 mm diameter at the center and corners of a 5 mm × 5 mm square) (Bagnoli sEMG system, Delsys Inc., United States) to record activities of the FDI muscle. By careful skin preparation and proper sensor application, the peak-to-peak value of baseline noise was controlled under 20 μV to secure the accuracy of EMG decomposition using EMG works v.4.1 (Delsys Inc., United States). The analog EMG signals from each pin-sensor were amplified (gain = 1,000) and filtered with a bandwidth of 20–450 Hz (De Luca et al., 2014). After that, four single differential EMG channels were obtained with pair-wise subtractions of the five pin-detections (voltages of the pin-sensor at the corner minus voltage of the pin-sensor at the center) (De Luca et al., 2006; Nawab et al., 2010; Hu et al., 2013). A high sampling rate of 20 KHz was used to avoid introducing phase skew across channels (De Luca et al., 2006, 2014; Nawab et al., 2010). Two active Ag-AgCl electrodes (3 mm diameter; Model F-E9M-40-5, Grass, United States) were placed 1 cm apart on the C3 area, which was over the hand area of the primary motor cortex. The reference electrodes for the EEG were placed on the bilateral earlobes. After amplification of the recorded signal (gain = 5,000), the EEG signal was hardware-filtered in the frequency range of 0.01–100 Hz and 60 Hz (Model P511, Grass, United States). Synchronized with the EMG system and force data, the EEG signal was sampled at 1,000 Hz.

### Stochastic Modeling of Force Fluctuation Dynamics

The force data used for behavior analysis were the RF data low-pass filtered at 20 Hz. To exclude force data irrelevant to visuo-motor processes and error correction (such as 8–12 Hz physiological tremor) (Slifkin et al., 2000; Vaillancourt et al., 2002), the RF was further conditioned with a digital low-pass filter (cut-off frequency: 6 Hz) (Chen et al., 2013; Lin et al., 2014). Then the conditioned force data in the time window of interest (8th to 37th second) were down-sampled to 100 Hz. The quality of the force-tracking performance was visualized with a return map for the time series of task errors, a graph of the task error *E*_i+1_ versus the previous task error *E*_i_ (Shenker, 1982; Mendez-Balbuena et al., 2012). A poor performance led to a dispersive distribution of error points in the map. In contrast, error points for a good performance concentrated near to the center of the map. The size of the task error was quantified with RMS of mismatch between target and force signal. In the temporal domain, RMS and SampEn were applied to calculate the size and complexity of Fc, defined as force data after removal of a linear trend (Hong and Newell, 2008). Fc characteristics reflect the degree of force steadiness and gradation strategy for force stabilization. SampEn is a popular and reliable entropy measure of the temporal aspects of biological variability (Richman and Moorman, 2000). The mathematical formula of sample entropy was SampEn $\left(m,\;r,\;N)=-\log(\frac{\sum_{i=1}^{N-m}A_{i}}{\sum_{i=1}^{N-m}B_{i}}\right)$, where *r* = 15% of the standard deviation of the force channel, *m* is the length of the template (*m* = 3), and *N* is the number of data points in the time series. *A*_i_ is the number of matches of the *i*th template of length *m + 1* data points, and *B*_i_ is the number of matches of the *i*th template of length *m* data points (Pethick et al., 2015). A larger value represents a more complex structure of the low-frequency Fc. In the spectral domain, the MF of Fc was determined based on the spectral profile estimated with a fast Fourier transform and the Welch method (Hanning window; window length: 2.048 s, overlapping time segment: 1/4 × window length) with a spectral resolution of 0.1 Hz. In addition, we quantified the spectral DOF, a statistic to reveal the power dispersion of Fc. Spectral DOF is calculated as DOF = ${\left(\sum_{i}^{N}S_{i}\right)}^{2}/\sum_{i}^{N}S_{i}^{2}$. The quantity is unity for a perfect single spectral peak, and a greater value of DOF represents a broader band of Fc (maximal value of *N* for white noise).

**FIGURE 2:**
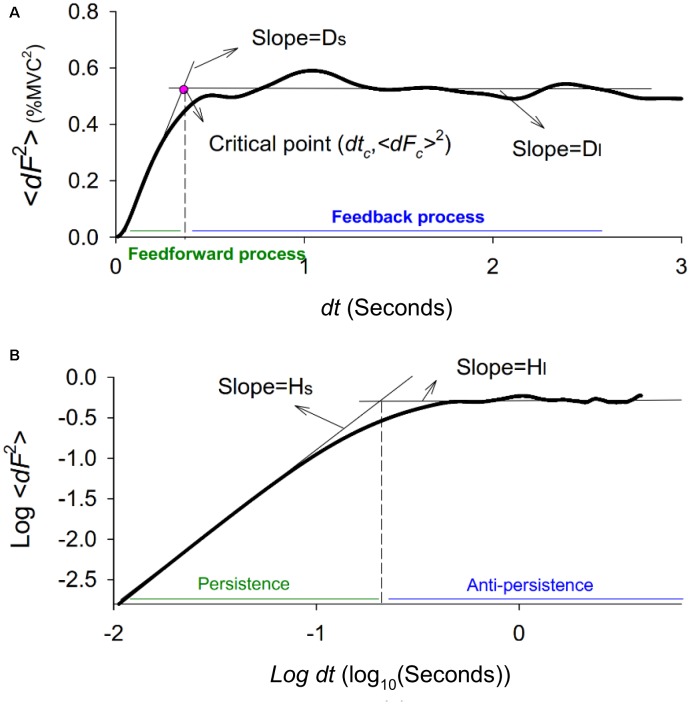
Stabilogram-diffusion plot. **(A)** A typical linear-linear stabilogram-diffusion plot. The short-term effective diffusion coefficient (*D*_s_), and long-term effective diffusion coefficients (*D*_l_) are regression slopes for the short time scale (0–0.5 s) and long time (0.5–3 s) scales. The critical point (*dt_c_*, <*dF_c_^2^*>) is the intersection point of the two regression lines, indexing a shift in open-loop and closed-loop control for the stochastic dynamics of force fluctuations. **(B)** A typical log-log stabilogram-diffusion plot. The computed short-term scaling exponent (*H*_s_) and long-term scaling exponent (*H*_l_) are regression slopes of the short time and long time scales of the log–log stabilogram-diffusion plot.

Force fluctuation dynamics were characterized with SDA, a probabilistic tool first proposed by Collins and De Luca (1993). The mathematical concept of the SDA approach was originally designated to resolve the statistical mechanics of a one-dimensional generalized family of Gaussian stochastic processes, such as postural sway (Collins and De Luca, 1993, 1995) and Fc (Chen et al., 2017a). The SDA describes the power-law relationship between the <*dF^2^*> and the *dt* in which these values occur; i.e., < dF^2^ >∼ dt^2H^. *H* is the scaling factor, a real number ranging from 0 to 1. For classic Brownian motion, *H* = 0.5. For the purpose of the present study, SDA was calculated by using the following equation: $\langle dF^{2}\rangle =\langle {\left[x(t+dt)-x(t)\right]}^{2}\rangle$, where <•> indicates the mean of the time series. The computation of *dF^2^* was empirically repeated with increasing *dt* values ranging from 0 to 3 s. The diffusion plot (linear–linear plots or log–log plots) was the mean square of Fc <*dF^2^*> against the time intervals *dt* (Figure 2A,B). Specifically, for biological systems regulated jointly by open-loop and closed-loop processes, the diffusion plots could be best-fitted with piecewise linear regression models, the cross-over phenomenon (Delignières et al., 2011). The *dt_c_* was the intersection of the two regression lines of the linear-linear diffusion plot (Figure 2A), and variations in the <*dF_c_^2^*> reflected a paradigm shift in force control (Collins and De Luca, 1993; Toosizadeh et al., 2015). In the linear–linear diffusion plot, the regression slopes (*D*_s_ and *D*_l_) of the short-term and long-term regions were two effective diffusion coefficients, which parameterized the control of the force stochastic activities in those regions, respectively. The *H*_s_ and *H*_l_ were linear fits of the log–log plot of the SDA (Figure 2B). A scaling exponent greater than 0.5 indicates that the system is governed by the open-loop process (persistence) and that the data series of the past and future are positively correlated (Collins and De Luca, 1993, 1995). Conversely, a scaling exponent smaller than 0.5 indicates that the data series of the past and future are negatively correlated, as regulated by the closed-loop process (anti-persistence). The selection of this model was a matter of physiological concern, due to the underlying shift in feedback and feedforward control for force stabilization with better use of the error information within the visual feedback.

### Probability of Motor Unit Discharge

**FIGURE 3:**
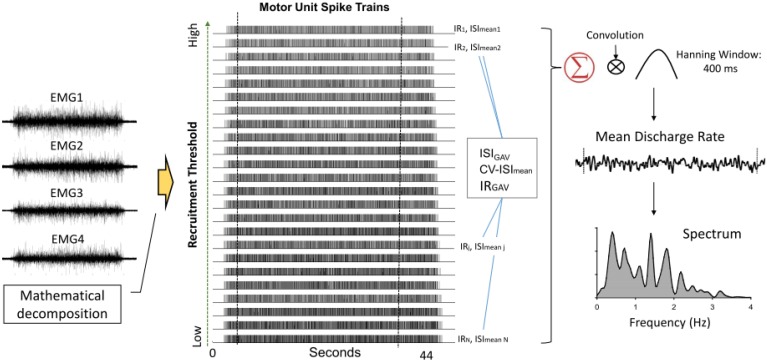
Acquisition of variables of inter-spike interval and mean discharge rate following mathematical decomposition of surface EMG into motor unit spike trains. Mean discharge interval (ISI_mean_) and irregularity index (IR) of each spike train are determined. The global averages of ISI_mean_ and IR, as well as coefficient of variance of ISI_mean_ (CV-ISI_mean_) among motor units (MUs). Mean discharge rate is obtained by smoothing the cumulative MU spike trains following convolution with a Hanning window (window length: 400 ms). Spectral distributions of mean discharge rate are estimated.

The action potential “templates” of MUs were decomposed from differential EMG channels using a previous proof-of-principle (De Luca et al., 2006; Nawab et al., 2010). Recent studies have shown that the artificial-intelligence-based computation algorithm can produce convincing decomposition results (Nawab et al., 2004; De Luca et al., 2015) via independent verification methods (Hu et al., 2013). The entire data collection period (44 s) was decomposed, resulting in binary spike trains that coded the activations of all MUs with values of 0 or 1 (Figure 3). Only discharge patterns of the window of interest were further analyzed. The validity of the EMG decomposition of each MU action potential train (MUAPT) was evaluated with the Decomposition-Synthesis-Decomposition-Compare (DSDC) test (De Luca et al., 1982, 2006). In brief, the DSDC test was used to decompose a synthetic sEMG signal, which was reconstructed by the summation of the predefined MUAPTs (or decomposed results) and Gaussian noise. The decomposed results were compared with the firing instances of predefined MUAPTs, and the percentage of the accuracy and location error of decomposition for each MUAPT was defined as decomposition accuracy. Previous studies have reported that the decomposition accuracy of MUAPTs ranges from 92.5 to 97.6% (De Luca et al., 1982, 2006). In this study, MUs of low decomposition accuracy (<90%) were excluded from the analysis. The discharge variables of MUs were determined in the time window of interest based on the decomposed EMG data of the overall 44 s. Three MU discharge variables were calculated, including global averaged inter-spike interval (ISI_GAV_), CV-ISI_mean_, and global averaged irregularity index (IR) of all MUs (IR_GAV_). In an experimental trial, ISI_mean_ was the mean value of all ISIs for an individual MUAPT, and the ISI_GAV_ was the averaged value of the ISI_mean_ for a group of MUs. Experimentally observed ISI variability among MUs was represented with the CV of the ISI_mean_ of a group of MUs (CV-ISI_mean_). Given a series of inter-spike intervals (ISI_i_) for a single MU, the irregularity index (IR) (Davies et al., 2006; Witham and Baker, 2007) is mathematically formulated as: $\mathrm{IR}=\frac{1}{N-1}\sum_{i=1}^{N-1}|\ln\;({\mathrm{ISI}}_{i+1}/{\mathrm{ISI}}_{i})|$. The IR_GAV_ was the averaged value of the IR for a group of MUs. An increase in force steadiness with LF-EA was likely associated with changes in inter-spike variability. The pooled behaviors of MU discharges were characterized with the mean discharge rate (Figure 3). To estimate the mean discharge trace, the global discharge rate was first determined by convolution of the cumulative spike trains of all the MUs with a Hanning window (window duration: 400 ms) (Hwang et al., 2017). The mean discharge rate was the global discharge rate divided by the number of detectable MUs in the experimental trial. The averaging process was used to standardize the amplitude of the global discharge rate across trials. Low-frequency oscillations of the mean discharge rate likely correspond to the common input to the motoneurons, providing a reasonable estimate of the force exerted by the muscle (Farina and Negro, 2015; Farina et al., 2016).

### Corticomuscular Coherence Estimation

**FIGURE 4:**
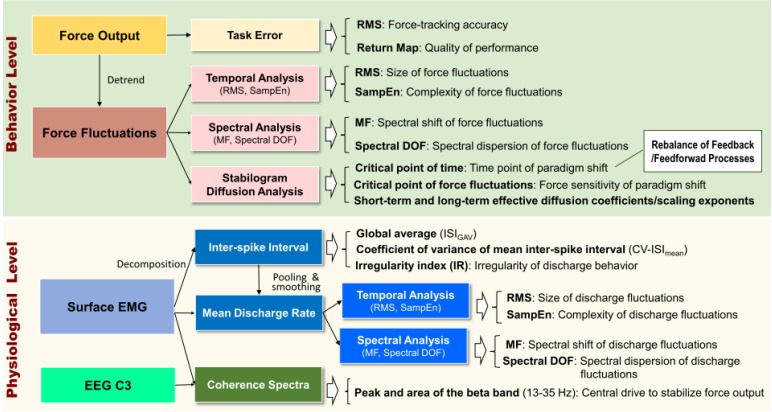
A summary diagram of behavior and physiological variables and their functional implications.

Corticomuscular coherence, especially in the spectral range of 13–35 Hz, is known to reflect efferent neural transmission to maintain force steadiness (Kristeva-Feige et al., 2002; Omlor et al., 2011). Four undecomposed EMG signals directly from differential channels were used to calculate CMC. The analog EMG signals were first resampled at 1 KHz, followed by signal conditioning with a band-pass filter (cut-off frequencies: 10 and 400 Hz). The conditioned EMG signal was rectified and high-pass filtered at 5 Hz (Chen et al., 2013). Ocular artifacts in the EEG recordings were removed. The EEG–EMG coherence was determined with EEG C3 and each conditioned EMG signal. The resulting EEG–EMG coherence spectra were averaged to represent the CMC of the experimental trial. The coherence between signals *x* and *y* at frequency *f, Coh_xy_(f)*, was determined according to the following equation: *Coh*_*xy*_(*f*) = $\frac{\left|S_{\mathrm{xy}}(f)\right|}{\sqrt{S_{\mathrm{xx}}(f)\times S_{yy}(f)}}$. The cross-spectrum between signals *x* and *y* at frequency *f* averaged across *N* data segments, *S_xy_(f)*, was calculated as follows: $S_{\mathrm{xy}}(f)=\frac{1}{N}\sum_{i=1}^{N}X_{i}(f)\times Y_{i}{\left(f\right)}^{*}$, where *X_i_ (f)* denotes the Fourier transform of the data segment *i* of the channel *x* at frequency *f*, and *Y_i_ (f)^∗^* denotes the complex conjugate of the Fourier transform of the data segment *i* of the channel *y* at frequency *f*. To estimate *Coh_xy_(f)*, EEG, and EMG signals were segmented into artifact-free epochs of 1.024 s without overlapping. Each segmented EEG and four EMG data from the differential channels were Hanning-windowed to minimize spectral leakage, and the *Coh_xy_(f)* of a given experimental trial was estimated with a total of 116 epochs (29 epochs/trial × 4 experimental trials). Spectral resolution was 1 Hz. The significance level of EEG–EMG coherence was the 95% CL. The CL was defined as: $\mathrm{CL}(\alpha )=1-{\left(1-\frac{\alpha }{100}\right)}^{1/N}$. Both the peak coherence and spectral area of the pooled EEG–EMG coherence spectrum in the beta band frequencies (13–35 Hz) were determined for each experimental trial, and those spectral variables of the three experimental trials were averaged for all the feedback conditions. All the behavior/physiological variables and their functional implications in this study are briefly summarized in Figure 4.

### Statistical Analysis

With reference to typical visual feedback to guide force-tracking, the primary research interest of this study was to contrast variations in the stochastic force behaviors and probability structure of MU discharges with the use of EA feedback and LF-EA feedback. On account of the relatively small sample size, the Wilcoxon signed-rank test was used to examine the task error, Fc variables (including SDA variables), inter-spike variables, variables of the mean discharge rate, and EEG–EMG coherence in the beta band in the three feedback conditions. The level of significance was 0.05. In the presence of significant main effects, *post hoc* testing was conducted using the Mann–Whitney *U* test with Bonferroni correction to determine the alpha level of significance (*p* = 0.0167). Spearman rank correlation was used to assess functional linkages between differences in task error between the EA/LF-EA and control conditions with the corresponding changes in those neurophysiological metrics that were sensitive to manipulation of EA. Signal processing and statistical analyses were completed in Matlab R2015b (Mathworks Inc., United States) and the statistical package for IBM SPSS software for Windows v.19.0 (IBM Inc., United States), respectively. Data reported in the text and figures without specific notations indicating otherwise are presented as mean ± standard error.

## Results

**FIGURE 5:**
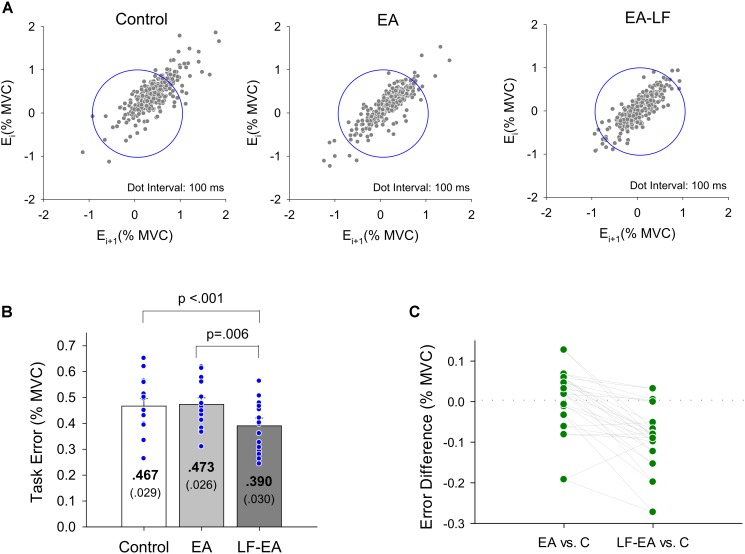
Task error properties. **(A)** Return maps of the force-tracking for a typical subject in the control, EA, and LF-EA condition. Graph of the error *E*_i+1_ versus previous error *E*_i_ where *i* is the sampling point. For brevity, the interval between two error data is set at 100 ms. Good performance of force-tracking exhibits error points that are concentrated near the center of the blue circle. **(B)** The contrasts of force-tracking error among the three visual feedback conditions. The blue dots represent force-tracking error of all individuals in this study. **(C)** A schematic plot to display scattering of differences in tracking error between the error amplification (EA)/low-frequency error amplification (LF-EA) and control (C) conditions. A more negative value of the error difference indicates a more positive performance benefit, underlying a relatively smaller tracking error in the EA or LF-EA condition. Each green dot represents EA-related differences in tracking error for an individual.

Figure 5A displays the return maps of the task errors from a typical subject in the three conditions: control, EA, and LF-EA. The dispersion of the error points in the maps for the LF-EA condition was smaller than those for the EA and control conditions. This was a qualitative way to characterize stable and accurate force-tracking with LF-EA. Figure 5B shows the population means, standard errors, and individual values of force-tracking errors for all three visual conditions. The results of the Wilcoxon signed-rank test revealed that force-tracking errors varied with feedback mode (χ_r_^2^ = 10.13, *p* = 0.006), with the smallest error for the LF-EA condition (*p* ≤ 0.006). Figure 5C shows the distribution of differences in tracking error between the EA/LF-EA and control conditions. The majority of the participants exhibited a more positive performance benefit with LF-EA than with EA, as indicated by the smaller mean tracking error relative to that of the control condition. Table 1 contrasts the differences in the Fc variables among the three visual conditions. The results revealed that all Fc variables were dependent on the feedback mode (*p* < 0.05). *Post hoc* analysis further revealed that the LF-EA condition exhibited the smallest RMS and the largest SampEn of Fc among the three feedback conditions (*p* < 0.01). Both the EA and the LF-EA conditions exhibited mean frequencies and spectral DOF larger than those of the control condition (*p* < 0.01). Functionally, LF-EA led to fine-grained and richer force gradation to rapidly remedy tracking deviations. In addition, the Fc dynamics were characterized with SDA, and all the SDA variables varied with manipulation of the feedback mode (*p* ≤ 0.006) (Table 2). *Post hoc* test indicated that *dt_c_* was smallest in the LF-EA condition and largest in the control condition (*p* < 0.01). In addition, <*dF_c_^2^*> was smaller in the LF-EA condition than in the control and EA conditions (*p* < 0.01). *D*_s_ and *H*_s_ were smallest in the LF-EA condition (*p* < 0.01), whereas *D*_l_ and *H*_l_ were largest in the LF-EA condition (*p* < 0.01). The observations indicated that the preclusion of high-frequency feedback components from the EA process led to task improvement during static force-tracking. The functional benefits were associated with the sensible detection of Fc (smallest *dt_c_* and <*dF_c_^2^*> in the LF-EA condition) and a shift in Fc control toward the feedback-prone process.

**Table 1:** Mean and standard errors of task error and force fluctuation variables for the control, error amplification (EA), and low-frequency error amplification (LF-EA).

Behavior variables (*n* = 15)	Control	EA	LF-EA	*Statistics*
Fc_RMS (% MVC)	0.424 ± 0.029^a^	0.424 ± 0.028^a^	0.376 ± 0.026^a^	χ_r_^2^ = 14.80, *p* = 0.001
Fc_SampEn	0.294 ± 0.017^b^	0.292 ± 0.016^b^	0.336 ± 0.023^b^	χ_r_^2^ = 11.02, *p* = 0.004
Fc_MF (Hz)	0.832 ± 0.049^c^	0.894 ± 0.040^c^	0.893 ± 0.045^c^	χ_r_^2^ = 12.13, *p* = 0.002
Spectra DOF	26.50 ± 1.40^c^	29.25 ± 1.05^c^	29.67 ± 1.27^c^	χ_r_^2^ = 6.40, *p* = 0.041


*^*a*^Control, EA > LF-EA, p < 0.01. ^*b*^LF-EA > Control, EA, p < 0.01. ^*c*^LF-EA, EA > Control, p < 0.01. (Fc, force fluctuations; RMS, root mean square; SampEn, sample entropy; MF, mean frequency; DOF, degree of freedom)*.

**Table 2:** Parameters of stabilogram diffusion analysis (SDA) of static force tracking in the control and error amplification (EA), low-frequency error amplification (LF-EA) conditions.

SDA variables (*n* = 15)	Control	EA	LF-EA	*Statistics*
*dt_c_* (s)	0.401 ± 0.016^a^	0.367 ± 0.015^a^	0.329 ± 0.014^a^	χ_r_^2^ = 20.93, *p* < 0.001
<*dF_c_*^2^> (%MVC^2^)	0.409 ± 0.063^b^	0.442 ± 0.071^b^	0.292 ± 0.040^b^	χ_r_^2^ = 14.80, *p* = 0.001
*D*_s_ (%MVC^2^/s)	0.601 ± 0.097^b^	0.682 ± 0.109^b^	0.419 ± 0.064^b^	χ_r_^2^ = 14.80, *p* = 0.001
*D*_l_ (%MVC^2^/s)	-0.014 ± 0.006^c^	-0.019 ± 0.008^c^	0.005 ± 0.002^c^	χ_r_^2^ = 14.53, *p* = 0.001
*H*_s_ (%MVC^2^/s)	0.940 ± 0.003^b^	0.942 ± 0.003^b^	0.937 ± 0.002^b^	χ_r_^2^ = 12.13, *p* = 0.002
*H*_l_ (%MVC^2^/s)	-0.088 ± 0.020^c^	-0.062 ± 0.014^c^	-0.015 ± 0.016^c^	χ_r_^2^ = 10.13, *p* = 0.006


*^*a*^Control > EA > LF-EA, *p* < 0.01. ^*b*^Control, EA > LF-EA, *p* < 0.01. ^*c*^LF-EA > Control, EA, *p* < 0.01. *(dt_*c*_, critical point of time; <dF_*c*_^*2*^>, critical point of force fluctuations; D_*s*_, short-term effective diffusion coefficients; D_*l*_, long-term effective diffusion coefficients; H_*s*_, short-term scaling exponent; H_*l*_, long-term scaling exponent)*.*

**FIGURE 6:**
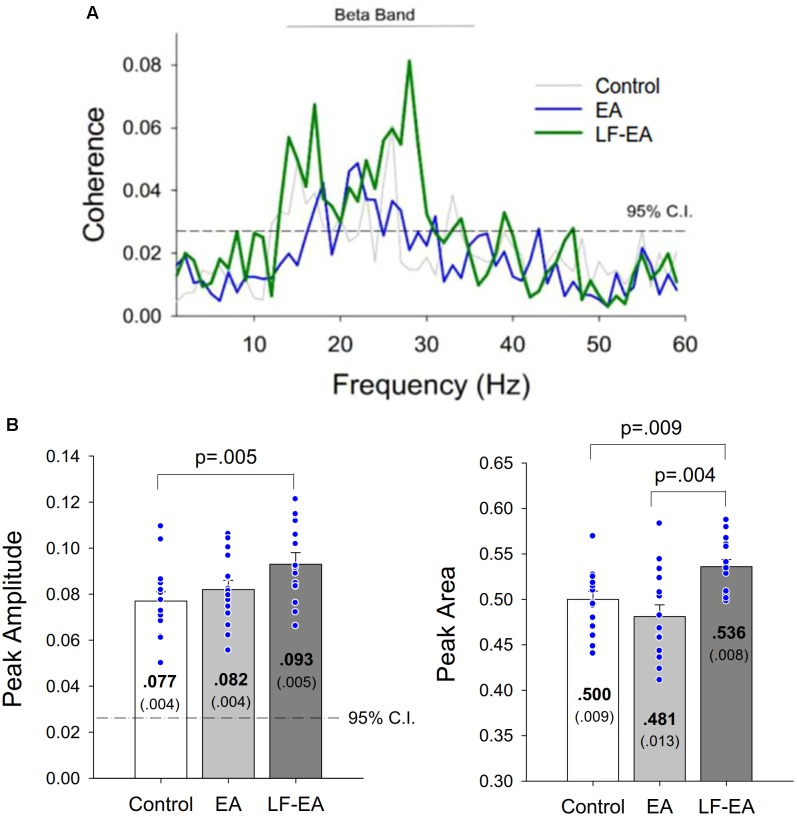
**(A)** Pooled coherence spectra between the EEG and rectified EMG of a typical participant in the control, error amplification (EA), and low-frequency error amplification (LF-EA) conditions. **(B)** the contrasts of peak coherence and spectral area in the beta band (13–35 Hz) among the three feedback conditions. The blue dots represent coherence values of all individuals in this study.

Under the condition of acceptable decomposition accuracy using the DSDC test (Control: 93.10 ± 0.42%; EA: 93.45 ± 0.42%; LF-EA: 93.76 ± 0.46%), the average numbers of analyzed MUs of an experimental trial did not vary with the feedback conditions (Control: 30.8 ± 1.8; EA: 32.1 ± 2.0; LF-EA: 31.5 ± 1.9; χ_r_^2^ = 1.97, *p* = 0.374), (χ_r_^2^ = 1.20, *p* = 0.549). Table 3A contrasts the inter-spike (ISI) variables of all MUs among the three feedback conditions. The global averages of the mean inter-spike interval (ISI_GAV_) and CV-ISI_mean_ were not affected by the feedback mode (*p* > 0.05). Only the discharge irregularity in terms of IR_GAV_ (or global average of IR for all MUs) varied significantly with feedback mode (*p* < 0.05). IR_GAV_ was generally highest in the LF-EA condition (*p* < 0.01). Table 3B contrasts the characteristics of the mean discharge rate of the all MUs among the three feedback conditions. Only the RMS of the mean discharge rate was subject to feedback mode (*p* = 0.005). *Post hoc* test revealed that the RMS of the mean discharge rate was significantly smaller in the LF-EA condition than in the control condition (*p* = 0.012). However, the SampEn, MF, and DOF of the mean discharge rate did not significantly vary with feedback mode (*p* > 0.05). Figure 6A presents an example of the pooled coherence spectra of the EEG and rectified EMG from a typical participant in the control, EA, and LF-EA conditions. The typical coherence spectra manifested with large power in the beta frequencies (13–35 Hz), exceeding the 95% CL. Figure 6B contrasts the population means of the peak coherence and spectral area in the beta frequencies among the three feedback conditions. Both the peak coherence (χ_r_^2^ = 7.60, *p* = 0.022) and the spectral area in the beta frequencies (χ_r_^2^ = 9.73, *p* = 0.008) varied significantly with feedback mode. Beta peak coherence was larger in the LF-EA condition than in the control condition (*p* = 0.005), and the spectral area in the beta frequencies was largest in the LF-EA condition (*p* ≤ 0.009). The use of LF-EA appeared to enhance CMC at 13–35 Hz, which might serve to stabilize the motor output and decrease the discharge variability.

**Table 3:** Means and standard errors of variables of inter-spike interval **(A)** mean discharge rate **(B)** from all motor units in the control, error amplification (EA), and low-frequency error amplification (LF-EA) conditions.

		Control	EA	LF-EA	*Statistics*
**(A) Discharge variables (*n* = 15)**	
	ISI_GAV_ (ms)	58.89 ± 3.15	58.78 ± 3.30	59.07 ± 2.99	*χ*_r_^2^ = 1.20, *p* = 0.549
	CV-ISI_mean_	0.238 ± 0.012	0.215 ± 0.012	0.222 ± 0.012	χ_r_^2^ = 5.20, *p* = 0.072
	IR_GAV_	0.198 ± 0.007^a^	0.194 ± 0.008^a^	0.208 ± 0.009^a^	χ_r_^2^ = 6.93, *p* = 0.031

**(B) Mean discharge rate (*n* = 15)**	
	RMS (Hz)	0.823 ± 0.067^b^	0.785 ± 0.065^b^	0.763 ± 0.057^b^	χ_r_^2^ = 10.53, *p* = 0.005
	SampEn	0.319 ± 0.009	0.308 ± 0.007	0.339 ± 0.007	χ_r_^2^ = 1.73, *p* = 0.420
	MF (Hz)	1.089 ± 0.025	1.074 ± 0.024	1.076 ± 0.017	χ_r_^2^ = 0.40, *p* = 0.819
	DOF	30.32 ± 0.88	29.88 ± 0.54	31.30 ± 0.85	χ_r_^2^ = 3.73, *p* = 0.155


*^*a*^LF-EA > EA, p = 0.023; LF-EA > Control, p < 0.01. ^*b*^Control > EA >, p = 0.023; Control > LF-EA, p = 0.012. (ISI_*GAV*_, global average of mean discharge interval (ISI_*mean*_) of all MUs; IR_*GAV*_, global average of irregularity index (IR); CV-ISI_*mean*_, Coefficient of variance of ISI_*mean*_ among motor units; RMS, root mean square; SampEn, sample entropy; MF, mean frequency; DOF, degree of freedom)*.

**FIGURE 7:**
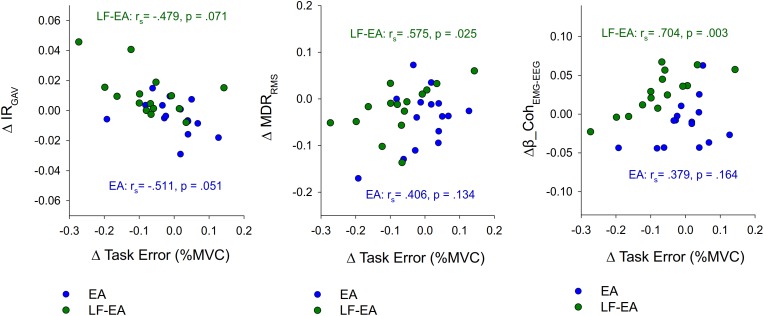
Scatter plots to show the relationships between EA-related changes in task error and neurophysiological metrics. Negative value of ΔTask Error represents task improve with EA or EA-LF feedback. (ΔTask Error, difference in task errors between the EA/LF-EA and control conditions; ΔIR_GAV_, differences in discharge irregularity between the EA/LF-EA and control conditions; ΔMDR_RMS_, differences in root mean square of mean discharge rate between the EA/LF-EA and control conditions; Δβ_Coh_EMG-EEG_, differences in beta-range EMG-EEG coherence area between the EA/LF-EA and control conditions; *r*_s_, Spearman rank correlation).

Figure 7 presents three scatterplots showing the associations between differences in task error and the neurophysiological metrics (ΔIR_GAV_, ΔMDR_RMS_, and Δβ-Coh_EMG-EEG_) sensitive to manipulation of EA. In terms of Spearman rank correlation (*r*_s_), the change in task error between the LF-EA and control conditions was significantly correlated to ΔMDR_RMS_ and Δβ-Coh_EMG-EEG_ (*p* < 0.05). In contrast, the change in task error between the EA and control conditions was not significantly correlated to ΔIR_GAV_, ΔMDR_RMS_, or Δβ-Coh_EMG-EEG_ (*p* > 0.05). These facts implied that task improvement in the LF-EA condition relative to that of the control condition could be linked to centrally mediated change in the amplitude of pooled discharges of the MUs.

## Discussion

The novel finding of this study was that gating of the high-frequency execution errors prior to virtual amplification (the LF-EA feedback) provided a functional benefit to the stabilization of static force, due to the smaller Fc with higher complexity, MF, and spectral DOF. The LF-EA feedback reduced the perceptual sensitivity to Fc (smaller <*dF_c_*^2^>) with a greater reliance on the visual feedback process for error corrections (smaller *dt_c_*). Physiologically, the shift in force control was associated with greater global discharge irregularity (IR_GAV_), smaller fluctuation in the mean discharge rate, and enhanced EMG-EEG coherence in the beta band.

### Structural Changes in Force Fluctuations and Implications for Force Control

The time series of Fc modeled with SDA was different from ordinary Brownian motion (un-correlated random-walk), as the diffusion curve of Brownian motion is linear and unbounded with the scaling exponent equal to 0.5 (Mandelbrot and van Ness, 1968; Collins and De Luca, 1993). The diffusion curve of the Fc changed slope after the critical point, and the scaling exponents for Fc were, respectively, greater than and less than 0.5 for short-term and long-term intervals (Figure 2B and Table 2). Hence, like postural sway (Collins and De Luca, 1993, 1995; Delignières et al., 2011), Fc are correlated and bounded random-walk signals, regulated distinctively by two subsystems. An open-loop process predominates Fc control in the short-term region with a scaling exponent greater than 0.5, for the stochastic activity was persistent and Fc data of the past and future were positively correlated. In contrast, a closed-loop process predominates Fc control in the long-term region. The stochastic activity with a scaling exponent smaller than 0.5 was anti-persistent, for Fc data of the past and future were negatively correlated (Collins and De Luca, 1993, 1995). This stochastic model of Fc is reminiscent of a continuum of the control regime of a visuomotor act ranging from feedback (closed-loop) to feedforward (open-loop) (Slifkin et al., 2000). Central to this interpretation is that the SDA variables of Fc in the LF-EA condition indicated a scheme switch of open- and closed-loop controls for static force control, as compared with those of the EA and control feedback modes. The smaller *dt_c_* and <*dF_c_^2^*> in the LF-EA condition (Table 2) reflected a drift in the equilibrium point of Fc control toward a closed-loop process (Kurz et al., 2013; Coubard et al., 2014; Toosizadeh et al., 2015). The interval of short-term stochastic activity governed by the open-loop regime (*dt_c_*) was significantly shortened, and feedback control was called into play when a smaller degree of Fc (<*dF_c_^2^*>) took place. The experimental observation was congruent with reductions in the *D*_s_ and scaling exponent (*H*_s_). After deconditioning of the feedforward mechanism, force-tracking in the LF-EA condition was more dependent on the feedback mechanism, with a functional benefit of superior task accuracy (Table 1). Hence, the prevailing use of the feedback process was conceptually in agreement with the perceptual narrowing (Easterbrook, 1959) and enhanced attentive control (Boussaoud and Kermadi, 1997; Jueptner and Weiller, 1998; Shirzad and Van der Loos, 2012) reported in behavioral studies.

Due to the smaller Fc with greater complexity (Table 1), the participants could develop fine-grained force-scaling with a richer correction strategy in the LF-EA condition with the feedback-prone process (Vaillancourt et al., 2002; Chen et al., 2013). Several lines of indirect evidence have shown that modulation of Fc dynamics in the LF-EA condition resembles characteristic changes in Fc after motor practice (Deutsch and Newell, 2004; Hwang et al., 2013). Moreover, the increase in the MF of Fc and flattening of the spectral DOF support of LF-EA indicated that the participants could increase the number of corrective attempts with abundant exploratory efforts to remedy tracking deviations. Gating the high-frequency components brought about these performance benefits because VEs above 0.8 Hz are too fast to be corrected. The interval to accomplish visuomotor correction in humans is at least 1 s (Navas and Stark, 1968; Miall et al., 1985), and primates cannot follow the full excursion of a target higher than 0.9 Hz with the feedback process (Miall et al., 1986). If visual EA contains information that cannot be rapidly responded with the feedback process, lag-induced feedback instability taxes attentional resources with processing visuomotor information that is irrelevant to task success. That is why the task accuracy, Fc properties, and SDA variables between EA and LF-EA were distinct.

### Variations in Motor Unit Discharge for Low-Frequency Error Amplification

The adaptation of the Fc dynamic originated from variations in the probability structures of the MU discharges. Physiologically, the decrease in the size of Fc with LF-EA was correspondent with the decrease in RMS of the mean discharge rate (Table 3B) rather than CV-ISI_mean_ (Table 3A). It is known that modeling of the mean discharge rate with a pooling process could accentuate synaptic inputs common to a population of active motoneurons but also attenuate the role of independent synaptic inputs to motoneurons (Farina and Negro, 2015; Farina et al., 2016). Therefore, the amplitude modulation of the mean discharge rate implies that LF-EA could effectively reduce the variations in the common input to a muscle. The observed influence of the common input confirms the model-based conjectures, implying a reduction in the intrinsic neuromotor noises at the motoneuronal level with EA (Wei et al., 2005; Hasson et al., 2016; Williams et al., 2016). The modulation of the size of the mean discharge rate was critical to the increase in task precision in the LF-EA condition (Figure 7). However, the reduction in the size of Fc with LF-EA is unlikely to have resulted from modulation of independent synaptic inputs to motoneurons because CV-ISI_mean_, which highlights the influence of synaptic inputs to motoneurons that differ from those that are common, was insensitive to feedback mode. On the other hand, the enhancement of the complexity of Fc in the LF-EA condition (Table 1) was nicely compatible with the irregularity of the increases in MU discharge (IR_GAV_) (Table 3A). However, the structures of the mean discharge rate, such as SampEn and DOF (Table 3B), did not well index the change in the complexity of Fc in the LF-EA condition. In addition to some unidentified organizational discharge activities, the viscous resistances of the musculotendon system attenuate the transmission of high-frequency neural drive to a muscle (Günther et al., 2007). This non-linearity often complicates the discharge–force relationship.

### Variation in Corticospinal Coupling for Low-Frequency Error Amplification

Instead, superior task accuracy and force steadiness in the LF-EA condition were associated with increased EEG–EMG coherence in the beta range (Figure 4). An increase in the beta-range EEG–EMG coherence represents greater synchronization of cortical activity to regulate common spinal inputs, a neural marker of steady-state motor output during static contraction (Perez et al., 2006; Kristeva et al., 2007). The beta-range CMC is greatly reduced when a force task is not steady (Salenius et al., 1997; Boonstra et al., 2009). Previous studies have reported that repetitive training can increase the precision of control in a static force task, in association with enhancement of beta-range CMC (Perez et al., 2006; Witte et al., 2007; Larsen et al., 2016). From all the neural sequelae, the enhanced beta-range CMC should contribute to a smaller size of discharge variability with enhanced complexity (Table 3 and Figure 7) and fine-grained force scaling with the feedback-prone process (Tables 1, 2) in the LF-EA condition. Since the beta-range corticomuscular rhythm is modifiable to peripheral sensory afferents (Riddle and Baker, 2005; Lalo et al., 2007), the precise force control in the LF-EA condition might be attributable to the reduction of the cognitive load of processing task-irrelevant error information, which would facilitate rapid integration of the visual and somatosensory information.

### Methodological Issues

A contrasting approach to enhance static force control is stochastic resonance (Mendez-Balbuena et al., 2012; Trenado et al., 2014). In addition to an increase in corticomuscular synchronization at 13–35 Hz, a better force precision with a return map of concentrated error points was noted following application of an optimal mechanical Gaussian noise. The task improvement was hypothesized to detect subthreshold sensory signals in the peripheral receptors, pertaining to noise-enhanced sensorimotor integration. However, stochastic resonance differs with the use of LF-EA, which minimizes cognitive load to process functionally irreverent noises. The return map with concentrated error points speaks for additional functional benefits for removal of high-frequency error components (noises) prior to EA (Figure 5A). Besides, one matter of concern is the decomposition of multi-electrode surface EMG. Although we cannot deny the likelihood of a small decomposition error (Piotrkiewicz and Türker, 2017), the state-of-the-art decomposition algorithm is a trade-off to capture the discharge variability among MUs and the force–discharge relation, based on a relatively large number of active MUs. To be rigorous, we applied a “reconstruct-and-test” procedure (Nawab et al., 2010; De Luca et al., 2015) to support the accuracy of the obtained identifications (91.2–97.1%) (De Luca et al., 2006; Nawab et al., 2010; Chen et al., 2017a,b; Hwang et al., 2017). The use of multi-channel surface EMG to explore MU behaviors has gained popularity in recent studies (Hu et al., 2014; Laine et al., 2015; Contessa et al., 2016; Chen et al., 2017a,b). In particular, the inconsistent changes in the complexity measures between IR_GAV_ and the SampEn of the mean discharge rate with LF-EA (Tables 3A,B) reinforce the role of decomposition in revealing diverse fractal myoelectric manifestations. A simulated EMG study showed that the fractal characteristic of surface EMG, which accounts for pooled MU behaviors, is jointly subject to variations in the CV of the discharge rate and the degree of MU synchronization (Mesin et al., 2016). Hence, fractal changes in the surface EMG are evident during fatiguing (Ravier et al., 2005) or higher-force (>25% MVC) contractions (Beretta-Piccoli et al., 2018). When the CV of the ISI is not expected to change, the discharge irregularity of a single MU such as IR_GAV_ could be masked by the interference pattern of surface EMG (or the mean discharge rate). Next, a low-pass filtering effect was likely to be effective only in the visual EA condition, though this study did not examine tracking performance in the non-EA condition. According to our preliminary study in healthy adults (*n* = 14), the task error of the control condition (0.467 ± 0.029% MVC) did not differ significantly from the task error in the condition of low-frequency feedback without EA (0.446 ± 0.039% MVC) (*t*_13_ = 0.648, *p* = 0.528) (unpublished data). Therefore, low-frequency error signals without amplification could not facilitate feedback control, and the performance benefit and paradigm shift were evident only in the LF-EA condition. Also, the selection of a low pass threshold of 0.8 Hz for EA was empirically determined. The time period necessary for the detection of visual information and motor adjustments was at least 150 ms (Miall et al., 1985, 1986), which prevented the participants from timely correcting fast-oscillatory error components. However, on account of the slow tracking response and perceptual motor conflict, the excessive removal of high-frequency error components is disadvantageous to task precision due to the lack of ample information for remedying tracking deviations. The effects of various low-pass thresholds on EA feedback will require further investigation.

## Conclusion

Virtual potentiation of low-frequency errors below 0.8 Hz for visual feedback more effectively improves task performance than does traditional EA or real visual feedback in a static isometric task. The selective gating of high-frequency error components reduces the task-irrelevant information in the visual feedback that cannot be rapidly processed with a feedback process. This study reveals that the amplification of low-frequency error information could increase the sensitivity to detect Fc and facilitate the state shift to the negative feedback process for force stabilization. The behavior adaptations arise from the promotion of effective corticospinal interactions to enhance discharge irregularity and minimize fluctuations of the common drive to a muscle.

## Author Contributions

I-SH and Y-CC: conception or design of the work. C-LH and Z-RY: acquisition. I-SH and C-LH: analysis. Y-CC, I-SH, and Y-TL: interpretation of data. I-SH and Y-CC: drafted the work or revised it critically for important intellectual content. Y-CC: final approval of the version to be published. Y-CC and I-SH: agreement to be accountable for all aspects of the work in ensuring that questions related to the accuracy or integrity of any part of the work are appropriately investigated and resolved.

## Conflict of Interest Statement

The authors declare that the research was conducted in the absence of any commercial or financial relationships that could be construed as a potential conflict of interest.

## Abbreviations

Δ MDR_RMS_: differences in root mean square of mean discharge rate between the EA/LF-EA and control conditions
Δβ_Coh_EMG-EEG_: differences in beta-range EMG-EEG coherence area between the EA/LF-EA and control conditions
ΔIR_GAV_: differences in discharge irregularity between the EA/LF-EA and control conditions
ΔTask Error: difference in task errors between the EA/LF-EA and control conditions
CL: confidence level
CMC: corticomuscular coherence
CV-ISI_mean_: coefficient of variance of mean ISI among motor units
<*dF^2^*>: mean-squared value of the force fluctuations
<*dF_c_^2^*>: critical point of force fluctuations
*D*_l_: long-term effective diffusion coefficient
DOF: degree of freedom
*D*_s_: short-term effective diffusion coefficient
DSDC: Decomposition-Synthesis-Decomposition-Compare test
*dt*: time interval
*dt_c_*: critical point of time
EA: error amplification
EEG: electroencephalography
EMG: electromyography
Fc: force fluctuations
FDI: first dorsal interosseus
*H*_l_: long-term scaling exponent
*H*_s_: short-term scaling exponent
IR: irregularity index of inter-spike interval
IR_GAV_: global average of irregularity index of inter-spike interval for all motor units
ISI: inter-spike interval
ISI_GAV_: global average of mean inter-spike interval for all motor units
ISI_mean_: mean value of inter-spike intervals in an individual MUAPT
LF-EA: low-frequency error amplification
MF: mean frequency
MU: motor unit
MUAPT: motor unit action potential train
MVC: maximal voluntary contraction
RE: real error
RF: real force
RMS: root mean square
SampEn: sample entropy
SDA: stabilogram diffusion analysis
T: target signal
VE: visualized error
VF: visualized force
